# Enhancing the Techno-Functionality of Pea Flour by Air Injection-Assisted Extrusion at Different Temperatures and Flour Particle Sizes

**DOI:** 10.3390/foods12040889

**Published:** 2023-02-19

**Authors:** Nasibeh Y. Sinaki, Jitendra Paliwal, Filiz Koksel

**Affiliations:** 1 Department of Food and Human Nutritional Sciences, University of Manitoba, 250 Ellis Building, 13 Freedman Crescent, Winnipeg, MB R3T 2N2, Canada; 2 Department of Biosystems Engineering, University of Manitoba, E2-376, EITC, 75A Chancellor’s Circle, Winnipeg, MB R3T 2N2, Canada

**Keywords:** yellow pea, food ingredients, starch gelatinization, techno-functional properties, extrusion, blowing agent

## Abstract

Industrial applications of pulses in various food products depend on pulse flour techno-functionality. To manipulate the techno-functional properties of yellow pea flour, the effects of flour particle size (small vs. large), extrusion temperature profile (120, 140 and 160 °C at the die) and air injection pressure (0, 150 and 300 kPa) during extrusion cooking were investigated. Extrusion cooking caused the denaturation of proteins and gelatinization of starch in the flour, which induced changes in the techno-functionality of the extruded flour (i.e., increased water solubility, water binding capacity and cold viscosity and decreased emulsion capacity, emulsion stability, and trough and final viscosities). In general, the large particle size flour required less energy input to be extruded and had higher emulsion stability and trough and final viscosities compared to the small particle size flour. Overall, among all of the treatments studied, extrudates produced with air injection at 140 and 160 °C had higher emulsion capacity and emulsion stability, making them relatively better suited food ingredients for emulsified foods (e.g., sausages). The results indicated air injection’s potential as a novel extrusion technique combined with modification of flour particle size distribution and extrusion processing conditions to effectively manipulate product techno-functionality and broaden the applications of pulse flours in the food industry.

## 1. Introduction

Techno-functional properties are the characteristics of a food ingredient that are important for its processing and technological applications. These properties are essential for determining the suitability of a food ingredient for specific food systems [1,2,3,4]. Techno-functional properties of the ingredients extracted from grains dictate their performance in industrial applications in various end-products including pasta, baked goods, snacks, etc. However, unlike the well-defined milling process and the strictly controlled flour particle size in the wheat flour industry (e.g., maximum particle size), pulse milling operations are not as widely studied and lack flour particle size criteria [5]. This complicates a valid comparison of the techno-functional properties of different pulse-based ingredients, and thus their applications in the food industry. The importance of a better understanding of the relationship between pulse milling, flour particle size and ingredient techno-functionality has previously been emphasized [6] and recently prompted a number of studies that investigated the complex relationship between the particle size distribution of grain flours and the techno-functional properties of the extruded ingredients made from them [7,8]. For instance, it was shown that the nitrogen solubility index of soybean meal extrudates increases with the average particle size of the soybean meal [8]. However, to date, there is no study on the effects of pulse flour particle size on the quality of the end-products made from them.

Extrusion has shown great potential to fine-tune ingredient techno-functionality due to its capability for precise control of the changes in food polymers (e.g., starch gelatinization, protein denaturation) as a function of temperature, pressure and shear during the process. For instance, it has been reported that extrusion cooking of pea and kidney bean flours [9] and yellow pea flour [10] increased their water holding capacity and water solubility. An increase in the emulsification properties as a result of extrusion has also been reported for extruded Mexican common bean flour [11], soybean protein-pectin blends [12], and soy protein concentrate [13].

Maintaining the renowned nutritional and environmental benefits of pulses warrants the investigation of novel technologies to further develop techno-functionally superior ingredients for use by the food industry. One such technology is blowing agent (e.g., N_2_, CO_2_ and air) assisted extrusion cooking [14,15,16,17,18]. Among different blowing agents, oxygen in the air can improve the techno-functional properties of protein-rich flours, as it can act as an oxidizing agent and convert free sulfhydryl groups of flour proteins to disulfides [19,20]. The potential of using oxidizing agents during extrusion was recently reported as a tool to improve techno-functional properties of yellow pea flour [17], where air injection-assisted extrusion resulted in extrudates with relatively higher water solubility, emulsion stability, and cold and trough viscosities.

Yellow pea flour is an attractive ingredient option for food manufacturers looking to add nutritional value to their products in a cost-effective manner [21,22]. Yellow peas contain relatively high levels of lysine, an essential amino acid that is often limited in cereal-based foods. In addition, yellow peas are known for their ability to fix nitrogen from the air, reducing the need for nitrogen fertilizers. This can result in a lower carbon footprint compared to other crops that require more synthetic fertilizers. These characteristics make yellow peas a competitive alternative ingredient for promoting sustainable agriculture and reducing the environmental impact of food production [21]. As a result, there have been numerous studies exploring their use in the manufacture of a wide range of food products, including snacks, cookies, and pasta, highlighting the potential of yellow pea flour as a valuable food ingredient that can contribute to a balanced diet [23]. This study builds on our previous work on the extrusion of yellow peas and aims to deepen the understanding of the impact of air injection during extrusion on the techno-functional properties of yellow pea flour. The focus is on the relationship between flour particle size, extrusion barrel temperature profile and the impact of air injection on the flour’s techno-functional properties. This study offers novel insights into the field of pulse flour extrusion, and contributes to the development of techno-functionally superior ingredients for the food industry.

## 2. Materials and Methods

### 2.1. Raw Material

Yellow pea (*Pisum sativum* L.) was obtained from AGT Food and Ingredients (St. Joseph, MB, Canada). The AC Agassiz variety of peas used in this study was grown in Southern Manitoba during the crop year of 2017. 

To produce pea flour of two different particle sizes, a rotor beater mill (Retsch SR 300, Retsch GmbH, Haan, Germany) with mesh sizes of (i) 0.75 mm to obtain flour with relatively smaller particle sizes (referred to as "small"), and (ii) 2.0 mm to obtain flour with relatively larger particle sizes (referred to as "large" throughout this study) was used. The particle size distribution of the small and large flours was measured using a particle size analyzer (Mastersizer 2000, Malvern Instruments Ltd., Worcestershire, UK). Particle size distribution of the raw flours was defined by d(0.1), d(0.5) and d(0.9), representing the size in microns below which 10%, 50% and 90% of the particles resided by volume, respectively. 

### 2.2. Proximate Composition Analyses

Moisture, protein, ash and lipid contents of flours were measured using AACC International standard methods 44-19.01, 46-30, 08-01 and 30-25, respectively [24]. Total carbohydrate content was calculated by subtracting protein, ash and lipid content from 1000.

### 2.3. Extrusion Process

A laboratory-scale co-rotating twin-screw extruder (MPF19, APV Baker Ltd., Peterborough, UK) with a circular die (diameter: 2.3 mm) was used at a screw speed of 220 rpm. Three different temperature profiles were employed along the five different zones of the extruder barrel extending from the feeder towards the die: 60–75–90–105–120 °C, 80–95–110–125–140 °C, and 100–115–130–145–160 °C. These temperature profiles are referred to as die temperature (DT) 120, 140 and 160 °C, respectively. The flour and water flow rates were kept constant at 2.5 kg h^−1^ and 0.22 kg h^−1^, respectively. For air-assisted extrusion, an injector valve located 22.9 cm before the die exit was utilized for the injection of pressurized air (Innovair Industrial Ltd., Winnipeg, MB, Canada) into the extruder barrel. A gas pressure regulator was used to set the air injection pressure at 150 or 300 kPa. Control extrudates were produced without air injection, i.e., 0 kPa. Torque and die pressure as extrusion process variables were recorded for each extrusion run. Specific mechanical energy (SME) during extrusion was calculated as described by Koksel and Masatcioglu (2018) [25] with a rated screw speed of 500 rpm and motor power of 2.2 kW. 

Extrudates were collected in duplicate at each temperature profile, dried in an air oven (Thermo Fisher Scientific, Heratherm Oven-OGS100, Dreieich, Germany) at 50 °C overnight and stored in zipped plastic bags until further analyses. The moisture content of extrudates after overnight drying was ≤10%. For techno-functionality and thermal analyses, extrudates were ground using a centrifugal mill (Retsch ZM 200, Retsch GmbH, Haan, Germany) with a sieve size of 250 µm.

### 2.4. Techno-Functional Properties

For measurements of water solubility (*WS*) and water binding capacity (*WBC*), 0.5 g raw or extruded flour was added to distilled water (10% *w*/*v*) and vortexed (Genie 2, Fisher Scientific, Asheville, NY, USA) at room temperature for 15 s, every 5 min, for 1 h. Then, the mixture was centrifuged (RC6 plus, Thermo Fisher Scientific, Asheville, NC, USA) at 5000× *g* for 10 min, and the supernatant was dried in an oven (Thermo Fisher Scientific, Heratherm Oven–Heratherm OGS100, Dreieich, Germany) at 100 °C. *WS* and *WBC* of the raw and extrudate flours were calculated using the following equations [26]:

(1) $$WS\;\left(\%\right)=\frac{\mathrm{Weight}\;\mathrm{of}\;\mathrm{dried}\;\mathrm{supernatant}\;}{\mathrm{Weight}\;\mathrm{of}\;\mathrm{extrudate}\;}\times 100$$

(2) $$WBC\;\left(\%\right)=\frac{\mathrm{Weight}\;\mathrm{of}\;\mathrm{wet}\;\mathrm{precipitate}-\;\mathrm{weight}\;\mathrm{of}\;\mathrm{dried}\;\mathrm{precipitate}}{\mathrm{Weight}\;\mathrm{of}\;\mathrm{extrudate}\;}\times 100$$

Emulsion capacity (*EC*) and emulsion stability (*ES*) of the raw or extruded flours were measured in triplicate using the method of Ahmedna, Prinyawiwatkul and Rao (1999) [27] with some modifications. In brief, 0.5 g raw or extruded flour was suspended in 5 mL distilled water. Each suspension was vortexed every 5 min for a total time of 30 min. Then, 3 mL of corn oil was added to each suspension to prepare the emulsions. The emulsions were homogenized for 2 min using a S18N-10G probe in an Ultraturrax homogenizer (T18 basic, IKA, Germany) and then centrifuged (RC6 plus, Thermo Fisher Scientific, Asheville, NC, USA) at 3000× *g* for 20 min. To obtain *ES*, each homogenized mixture was incubated in a water bath at 45 °C for 30 min, cooled down to 25 °C for 20 min and then centrifuged at conditions similar to those of the *EC* test. A digital caliper (accuracy 0.01 mm) was used to measure the height of the emulsified phase and the height of the whole mixture, the ratio of which was used to define *EC* and *ES*. 

A Rapid Visco Analyzer (RVA 4, Newport Scientific, Warriewood, Australia) was used to measure the pasting properties of the raw and extruded flours following the method of Li, Masatcioglu and Koksel (2019) [16]. Extruded flours (3.5 g, 14% mb) were mixed with 25 mL distilled water to prepare a slurry whose viscosity was measured during the following conditions: keep at 25 °C for 2 min, heat to 95 °C at a speed of 14 °C min^−1^, hold at 95 °C for 3 min, cool down to 25 °C, hold at 25 °C for 5 min.

### 2.5. Thermal Properties

Thermal properties of a select set of raw and extruded flours were analyzed using a differential scanning calorimeter (DSC Q series 200, TA Instruments, New Castle, DE, USA). Briefly, ~10 mg of raw (or extruded) flour was weighed into an aluminum DSC pan, followed by the addition of deionized water (40 µL). The pan was hermetically sealed and stored for 48 h at 4 °C for equilibration. The equilibrated pan was placed in a standard DSC cell with an empty pan as a reference and heated at a rate of 5 °C min^−1^ from 25 to 125 °C. Onset temperature (To), peak temperature (Tp) and enthalpy (ΔH) were calculated using the TA Universal Analysis Software (version 4.5A, TA Instruments, New Castle, DE, USA). Results were reported as means of triplicate analyses.

### 2.6. Statistical Analysis

Techno-functional and thermal properties were evaluated by one-way analysis of variance (ANOVA) and Tukey pairwise comparison test using Minitab Statistical Software (Version 17, Minitab Corp., State College, PA, USA) at a 95% confidence interval (*p* ≤ 0.05). Pearson correlation was also carried out to determine correlation coefficients between extrusion variables and various techno-functional and thermal properties of extruded yellow pea flour.

## 3. Results and Discussion

### 3.1. Particle Size and Proximate Composition

**Figure 1:**
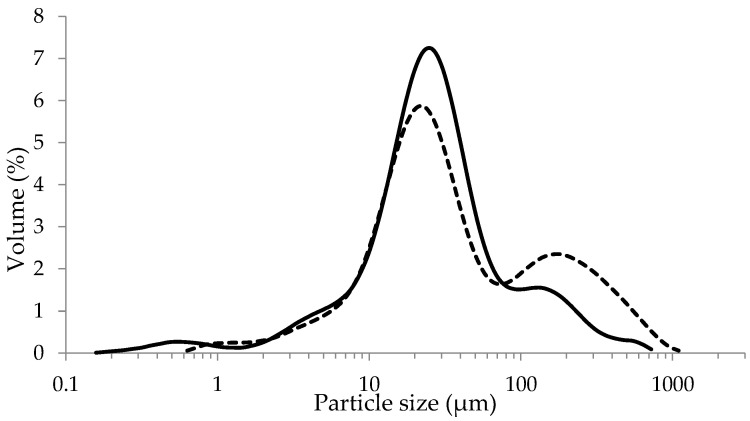
Particle size distribution of yellow pea flours milled with mesh sizes of 0.75 mm (solid curve) and 2.0 mm (dashed curve).

The particle size distribution of the raw flours is shown in Figure 1. The d(0.1), d(0.5) and d(0.9) values for the small particle size flour were 6.4 µm, 23.7 µm and 114.0 µm, respectively, whereas these values were 8.8 µm, 31.1 µm and 296.1 µm, respectively, for the large particle size flour. As expected, d(0.1), d(0.5) and d(0.9) values were higher in the large particle size flour compared to the small particle size flour, with d(0.9) being more than double and significantly higher (*p* < 0.05) for the large particle size flour. The extent of the difference in d(0.1) and d(0.5) was not as dramatic for the two flours. As the average size of a flour particle increases, its total surface area to volume ratio decreases. Consequently, water penetration and diffusion to the center of the particles was expected to be slower for flour with relatively larger size particles. 

The small particle size flour contained 212.2 ± 2.4 g protein, 26.4 ± 0.0 g ash, 16.3 ± 0.1 g lipid and 745.1 ± 2.4 g total carbohydrates per kg of dry flour, while the large particle size flour contained 222.7 ± 2.6 g protein, 27.0 ± 0.1 ash, 16.3 ± 0.1 g lipid and 734.1 ± 2.5 g total carbohydrates per kg of dry flour. Based on these results, small and large particle size flours had similar lipid contents, but the large particle size flour had higher (*p* < 0.05) protein and ash content, and also lower total carbohydrates (by difference) content compared to the small particle size flour. These findings suggest that milling and grinding operations may impact the proximate composition of the flour, likely through their effects on flour yield. A recent study on roller milling of yellow peas and red lentils revealed that the mean flour particle size significantly affected the milling yield and the proximate composition of the flours produced [28]. Similarly, Maskus et al. (2016) [29], who studied the effect of different grinding systems (e.g., stone milling and hammer milling) on flour particle size, reported that whole yellow pea flours with higher mean particle size had higher protein content than the ones with lower mean particle size. Further research is needed to fully understand the reason for the slight differences observed for the protein and carbohydrate contents in different particle size flours and the relationship between milling operations, flour particle size and flour proximate composition. 

### 3.2. Extrusion Process Variables

The effects of extruding yellow pea flour at different particle sizes, die temperatures and air injection pressures on torque, die pressure and specific mechanical energy (SME) values are presented in Table 1. In general, torque, die pressure and SME values were similar or higher during the extrusion of the small particle size flour compared to those of the large particle size flour. Based on the relatively lower total surface area to volume ratio of large particle size flour compared to that of small particle size flour, slower water penetration and diffusion to the center of the larger particles are expected to limit starch gelatinization [7,30,31,32,33]. A lower level of starch gelatinization results in a lower melt viscosity [32], which is reflected in lower torque and SME values during extrusion, in line with our findings. In general, torque, die pressure and SME decreased with increasing temperature, possibly due to a decrease in dough viscosity at higher temperatures [15,34]. In line with this finding, a highly significant and negative correlation was observed between die pressure and extruder die temperature (r = −0.8512, *p* < 0.001). Overall, air injection increased the die pressure but decreased the torque and SME values compared to extrusion without air injection, suggesting that air injection acted as a plasticizer due to its possible partial dissolution into the melt inside the barrel under pressure. Similarly, decreases in torque and SME values with gas injection were reported by Sinaki et al. (2021) [17] for air injection during the extrusion of yellow pea flour. 

### 3.3. Water Solubility (WS) and Water Binding Capacity (WBC)

**Figure 2:**
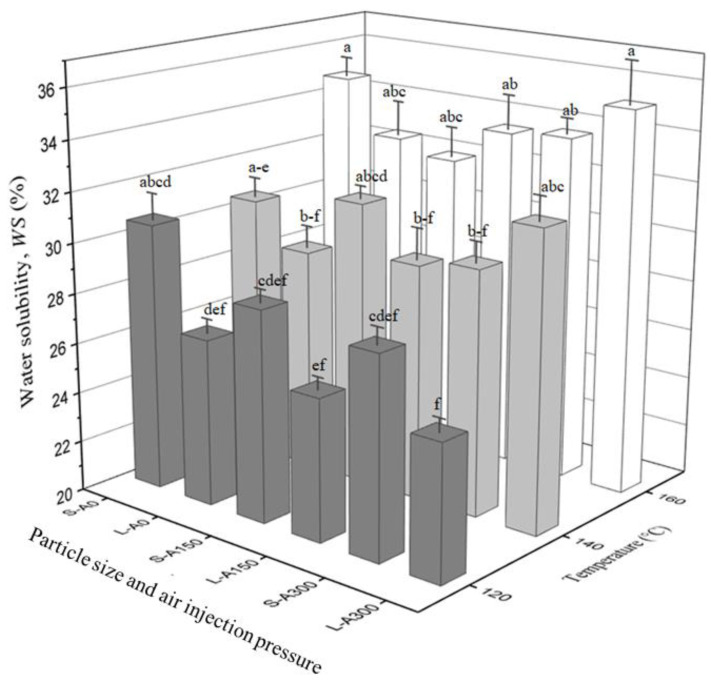
Water solubility (*WS*) values of yellow pea extrudates produced with air injection at 0 kPa, 150 kPa and 300 kPa, i.e., A0, A150 and A300, and two different flour particle sizes, i.e., small (S) and large (L), as a function of die temperature. Values marked with different letters are significantly different (*p* ≤ 0.05). Error bars represent ± standard error (*n* = 4).

**Figure 3:**
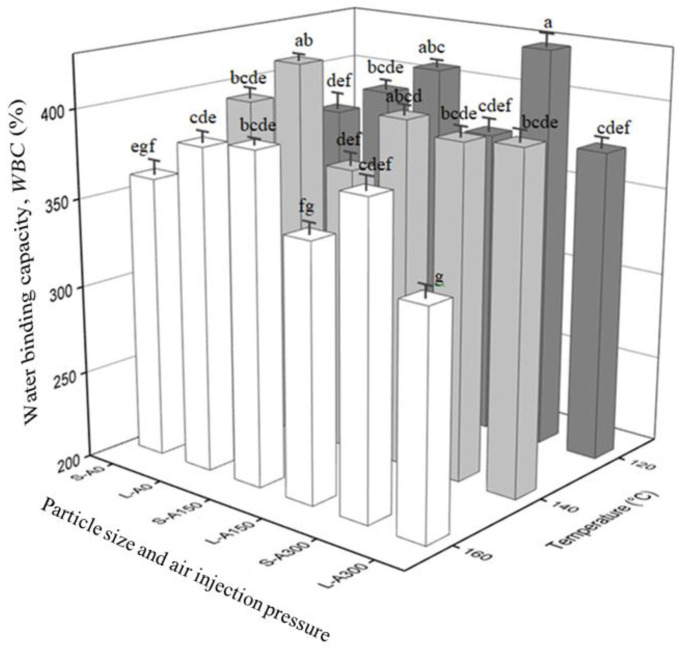
Water binding capacity (*WBC*) values of yellow pea extrudates produced with air injection at 0 kPa, 150 kPa and 300 kPa, i.e., A0, A150 and A300, and two different flour particle sizes, i.e., small (S) and large (L), as a function of die temperature. Values marked with different letters are significantly different (*p* ≤ 0.05). Error bars represent ± standard error (*n* = 4).

*WS* and *WBC* of extrudates as a function of flour particle size, die temperature and air injection pressure are shown in Figure 2 and Figure 3, respectively. *WS* and *WBC* of raw yellow pea flour at both flour particle sizes (*WS* of 20.4% and *WBC* of 160.5% for the small particle size flour; and *WS* of 20.9% and *WBC* of 165.8% for the large particle size flour) were significantly lower than those of their respective extrudates, showing that extrusion cooking improved *WS* and *WBC*. These findings are consistent with reports in the literature that extrusion enhances flour’s *WS* and *WBC*, as also reported for pea and kidney bean flours [9], African breadfruit, soybean and corn flours [35], yellow pea flour [10], and corn gluten meal [36]. 

In general, there was a trend for extrudates produced with the small particle size flour to have similar or higher *WS* values compared to those produced with the large particle size flour. Although statistically insignificant, the increase in *WS* with decreasing particle size can be attributed to an increase in the ratio of the surface area of particles to unit volume [7], a higher level of starch gelatinization and/or melting [37] during extrusion, and thus more amylose and amylopectin fragments leaching out of starch granules [30,32,38]. When the effects of die temperature were considered, *WS* values for extrudates produced at 160 °C were at least as high as those that were produced at 120 °C (Figure 2). Correlation analysis showed that a highly significant and positive correlation existed between *WS* and die temperature (r = 0.8800, *p* < 0.001). Increasing the die temperature increases starch gelatinization and/or melting and, therefore, *WS* values [38]. No consistent trend regarding the impact of air injection during extrusion cooking on *WS* values was observed, indicating the complexity of the changes in partial gas dissolution as a function of die temperature and how flour particle size affects the pressure build-up inside the barrel and at the die (Table 1).

The *WBC* of extrudates was not significantly (*p* ≤ 0.05) affected by the flour particle size. The only exceptions to this trend were the extrudates produced at 120 °C and air injection at 300 kPa and the extrudates produced at 160 °C and air injection at 150 and 300 kPa, where the extrudates made from the smaller particle size flour had significantly (*p* ≤ 0.05) higher *WBC* compared to those made from large particle size flour. The fact that the *WBC* of extrudates made from small particle size flour improved only in the presence of the blowing agent indicated a mechanism that involves air, specifically oxygen due to its relatively higher solubility in water when compared to nitrogen [39]. Oxygen present in air may act as an oxidizing agent, causing oxidation of flour proteins and promoting the formation of disulfide bonds among protein polymers [40,41,42,43,44,45]. Consequently, protein–water interactions may decrease, increasing the availability of water to interact with the starch [46,47], resulting in a higher level of starch gelatinization and/or melting, exposure of starch’s hydroxyl groups to water molecules, and thus an increase in *WBC* of the extrudates produced with air injection. The increased starch gelatinization levels in extrudates produced by air injection were confirmed by thermal analysis. In Table 2, a comparison of the starch gelatinization peak characteristics for the extrudates made from the small particle size flour as a function of air injection pressure is presented. The results indicated that air injection pressure had a significant effect on the level of starch gelatinization, i.e., as air injection pressure increased, the starch gelatinization enthalpy decreased and was significantly lower for extrudates produced with air injection at 300 kPa compared to those produced with no air injection. Similar results were observed for the rest of the extrudates as a function of air injection pressure (data not shown). Given air injection’s impact on the degree of starch gelatinization, these findings suggest that air injection can potentially be used to replace relatively more intense extrusion conditions to achieve similar starch gelatinization levels.

### 3.4. Emulsion Capacity (EC) and Emulsion Stability (ES)

Emulsion capacity and emulsion stability refer to the ability of a material, a food ingredient in this case, to form and stabilize emulsions. *EC* and *ES* values of extrudates as a function of flour particle size, die temperature and air injection pressure are presented in Table 3. *EC* and *ES* values of raw yellow pea flour at both flour particle sizes were significantly higher than those of their respective extrudates, showing that extrusion cooking lowered *EC* and *ES*. These findings are consistent with reports in the literature that extrusion decreases *EC* and *ES* of the starting material, as was also reported for soybean meal [8], chickpea-sorghum and chickpea-maize flours [48], chickpea and sorghum flour [49], yellow pea flour [10], and rice flour [50]. The decrease in *EC* and *ES* can be due to a reduction in protein solubility after extrusion cooking, as protein solubility is positively correlated with emulsifying properties of proteins [8,51,52,53]. Our results also showed that when the intensity of extrusion increased (as measured by SME), *ES* decreased (r = −0.7340 between *ES* and SME, *p* ≤ 0.05), suggesting a decrease in protein solubility at higher SME values.

In terms of the effect of the particle size on the *EC* and *ES* of the raw flours, the results showed that the raw small particle size flour had higher *EC* and *ES* values compared to the raw large particle size flour, similarly to the results previously reported for rice flour [50] and yellow pea flour [46]. It has been argued that the reduction in the particle size of the flour increases the *EC* and *ES* due to an improvement in the dispersion of its emulsifying components such as proteins, causing higher interactions of these components with the oil–water interface [46]. 

The *EC* values of the extruded small and large particle size flours were similar, meaning that flour particle size was not a significant factor affecting the *EC* of the extrudates. The only exceptions to this trend were the extrudates produced at 120 °C without air injection, where the extrudates made from the small particle size flour had significantly (*p* ≤ 0.05) higher *EC* compared to those made from the large particle size flour. When the effects of die temperature were considered, in general, extrudates produced at 160 °C showed higher *EC* values compared to those produced at 120 °C (Table 3). In line with this, a moderate and significant correlation between the die temperature and the *EC* values of the extruded flours was found (r = 0.5570, *p* ≤ 0.05). Similarly, Martínez et al. (2014) [50] showed that increasing extrusion temperature enhanced the emulsion capacity of rice flour. The only exceptions to this trend were the extrudates produced with small particle size flour without air injection, where the extrudates produced at 120 °C had significantly (*p* ≤ 0.05) higher *EC* compared to those at 140 °C and 160 °C. The higher *EC* at a higher temperature may be attributed to the aggregation of protein molecules at a higher level due to enhanced protein–protein and protein–oil interactions at higher temperatures [50,54]. Additionally, an increased level of starch gelatinization at high temperatures may be responsible for the higher *EC* of these extrudates, as gelatinized starch can act as a protective barrier between oil droplets in an emulsion [46,55,56]. The effect of air injection on the *EC* of extrudates was not significant (*p* ≤ 0.05) for most of the extrudates except for those produced with the small particle size flour at 120 °C, for which air injection significantly (*p* ≤ 0.05) decreased *EC*. It is possible that air injection at 120 °C reduced protein solubility, leading to a decrease in *EC*. However, a comprehensive investigation of the protein structural modifications is outside the scope of this study and can be explored further in a future study. 

Based on Table 3, the *ES* values of the extruded large particle size flour produced at 120 and 140 °C were significantly (*p* ≤ 0.05) higher compared to those of the extrudates produced with small particle size at 120 and 140 °C, meaning that flour particle size was a significant factor affecting *ES*. In line with this finding, a positive correlation was observed between *ES* and flour particle size (r = 0.5670, *p* ≤ 0.05). When the effects of die temperature were considered, in general, extrudates produced at 160 °C showed higher *ES* values compared to those produced at 120 °C (Table 3). Correlation analysis showed that a positive correlation existed between *ES* and die temperature (r = 0.5850, *p* ≤ 0.05). The increasing temperature may result in changes in protein structure that produce relatively more flexible proteins (e.g., an increase in random coil structures) that can be more easily and rapidly adsorbed at the oil interface, resulting in higher *ES* values [13,50]. The effect of air injection on *ES* values was air injection pressure- and flour particle size-dependent. Air injection at 300 kPa significantly (*p* ≤ 0.05) increased *ES* values of extrudates produced from the large particle size flour, while air injection could not significantly (*p* ≤ 0.05) affect *ES* values of extrudates produced from the small particle size flour. It is possible that with air injection, a higher dissolved oxygen concentration in the melt may have caused protein aggregation and exposure of originally buried hydrophobic sites of proteins, causing an increase in *ES*. Our results suggest that manipulation of extrusion conditions, especially air injection during extrusion cooking of the large particle size yellow pea flour, can be used to produce ingredients with better *ES*, and, therefore, better fit for applications including meat extenders for sausages, burger patties, frankfurters, etc.

### 3.5. Pasting Properties 

The pasting properties of extrudates as a function of flour particle size, die temperature and air injection pressure are shown in Table 4. Before extrusion, the small particle size flour had higher cold, peak, trough and final viscosities compared to the large particle size flour. These results are in agreement with Maskus et al. (2016) [29], who studied the pasting properties of whole and split yellow pea flours with different particle sizes. 

Extrusion cooking caused significant changes in the pasting properties of the flours. Extrusion resulted in an increase in the cold viscosity due to the starch gelatinization/melting that occurs during extrusion cooking [57]. The extrusion also resulted in a reduction in the trough and final viscosities, in line with the results of Wang, Ai, et al. (2019) [49], who studied the pasting properties of chickpea flour extruded at similar temperatures. An overall reduction of the viscosity values in the pasting curve after extrusion indicated that starch granules are disrupted to a greater extent in extruded flour compared to raw flour. During RVA testing, the amylose and amylopectin that leach out of starch granules during extrusion align in the direction of the mixing action of the paddles, causing lower trough and final viscosities [58,59]. Raw flour peak viscosity, independent of its particle size, was around 95 °C, while the extruded flours did not show a peak in their viscosity profile, possibly as a result of the extensive starch degradation during the extrusion cooking [25]. These findings are in line with the results of the thermal analysis, namely that the gelatinization enthalpy of the raw flour was almost 10× higher than those of the extrudates (starch gelatinization enthalpy of 3.14 ± 0.08 J/g for the raw flour with small particle size vs. 0.38 ± 0.04 J/g on average for the small particle size flour extruded at a die temperature of 120 °C).

In general, extrudates produced at 140 and 160 °C showed higher cold, trough and final viscosities compared to those produced at 120 °C. In line with this, a moderate and significant correlation between the die temperature and the cold viscosity of the extruded flours was found (r = 0.5085, *p* ≤ 0.05). Similar results have been reported by Wang, Nosworthy, et al. (2019) [48], who showed that trough and final viscosities of chickpea-sorghum and chickpea-corn extrudates increased with increasing die temperature from 120 to 150 °C due to the increase in starch gelatinization during the extrusion. Extrudates produced with large particle size flour had higher trough and final viscosities compared to the extrudates produced with small particle size flour under all extrusion conditions studied (Table 4).

It was clear that the interactive effects of flour particle size and air injection pressure were complex. For example, air-injected extrudates produced with small particle size flour at lower die temperatures of 120 and 140 °C in general had higher cold, trough and final viscosities compared to those produced at the highest die temperature of 160 °C. However, for the air-injected extrudates with large particle sizes, the trend was different. These extrudates had lower cold and trough and higher final viscosity values when compared to those produced with no air injection. The reason for differences in RVA viscosities at different air injection pressures and die temperatures in large and small particle-size flours is attributed to the presence of different levels of degraded starch in their counterpart extruded flours. A higher level of starch degradation might occur as a result of air injection into the extruder barrel, as confirmed by thermal analysis (Table 2), which is likely due to a localized increase in the pressure inside the extruder barrel during air injection-assisted extrusion in the presence of additional gas. This was visible for most treatments in this study, as reported in Table 1. 

## 4. Conclusions

The utilization of pulses in the food industry can be broadened by improving their techno-functional properties. However, several factors, such as pulse flour particle size, lead to variability in the techno-functionality of different pulses in various food applications. To better understand the complexity that particle size variation brings to yellow pea flour techno-functionality, this study focused on its relationship to temperature profile and air injection pressure during extrusion cooking. An increase in raw flour particle size generally caused a decrease in torque, die pressure and SME during extrusion, meaning that larger particle-size flour required less mechanical energy input to be processed. Overall, water solubility was the highest for the extrudates produced at the highest die temperature and extrudates made from small particle flour, making them more suitable for products such as sports drinks and protein shakes. In general, extrudates produced with air injection and at the higher extrusion temperature had higher emulsion capacity and stability, making them more suitable for extending meat products such as burger patties and sausages. The impact of air injection was dependent on the feed particle size, extrusion temperature and injection pressure. Overall, the injection of air at 300 kPa had a higher impact on techno-functional properties compared to that at 150 kPa or no air injection. The results of this study indicate that a novel extrusion technique (i.e., air injection-assisted extrusion cooking) combined with conventional modifications such as feed characteristics (e.g., particle size distribution) and processing conditions (e.g., temperature profile) can be used to effectively manipulate techno-functional properties of extruded pulse flours. Future work will focus on changes in protein structure with extrusion and how these changes affect ingredient techno-functionality.

## Figures and Tables

**Table 1:** Experimental design and dependent extrusion process variables.

Flour Particle Size	Die Temperature (°C)	Air Injection Pressure (kPa)	Torque (%)	Die Pressure (kPa)	SME * (Wh kg^−1^)
Small	120	0	35 ± 0 a	6500 ± 100 cd	319 ± 2 a
		150	31 ± 0 b	6950 ± 250 bc	285 ± 2 b
		300	29 ± 1 c	7650 ± 150 a	263 ± 12 c
	140	0	29 ± 0 c	5250 ± 150 e	263 ± 4 c
		150	23 ± 0 ef	6000 ± 0 d	215 ± 2 ef
		300	23 ± 0 ef	6600 ± 0 bc	213 ± 0 ef
	160	0	25 ± 0 de	5150 ± 50 e	227 ± 2 de
		150	24 ± 0 de	4600 ± 0 fg	221 ± 0 de
		300	24 ± 0 de	4850 ± 50 ef	223 ± 2 de
Large	120	0	25 ± 0 de	6700 ± 100 bc	230 ± 2 de
		150	25 ± 0 de	6450 ± 50 cd	230 ± 2 de
		300	26 ± 0 d	7100 ± 0 b	236 ± 0 d
	140	0	21 ± 0 f	5100 ± 0 ef	196 ± 6 f
		150	17 ± 0 gh	5000 ± 0 ef	155 ± 0 gh
		300	14 ± 0 i	4150 ± 50 gh	126 ± 2 i
	160	0	18 ± 0 g	3100 ± 0 i	165 ± 2 g
		150	15 ± 0 hi	3950 ± 50 h	138 ± 2 hi
		300	18 ± 0 g	4250 ± 50 gh	165 ± 2 g

* SME: Specific mechanical energy. Torque, die pressure and SME values in a column are significantly different if designated with different letters (*p* ≤ 0.05). Values represent mean ± standard error (*n* = 2).

**Table 2:** Thermal properties of extruded yellow pea flours produced at a die temperature of 120 °C with small flour particle size and as a function of air injection pressure.

Air Injection Pressure (kPa)	To (°C)	Tp (°C)	∆H (J/g)
0	80.08 ± 0.21 a	85.06 ± 0.28 a	0.41 ± 0.01 a
150	80.08 ± 0.11 a	85.17 ± 0.04 a	0.40 ± 0.01 ab
300	80.12 ± 0.18 a	85.08 ± 0.14 a	0.34 ± 0.02 b

To: Onset temperature; Tp: Peak temperature; ∆H: Enthalpy of starch gelatinization. Thermal properties of extruded flours in a column are significantly different if designated with different letters (*p* ≤ 0.05). Values represent mean ± standard error (*n* = 3).

**Table 3:** Emulsion capacity (*EC*) and emulsion stability (*ES*) of raw and extruded yellow pea flours as a function of flour particle size, die temperature (DT) and air injection pressure.

Flour Particle Size	DT (°C)	Air Injection Pressure (kPa)	*EC* (%)	*ES* (%)
Small-Raw	-	-	33.9 ± 0.7 a	36.8 ± 0.5 a
Small-Extruded	120	0	31.2 ± 0.2 b	25.9 ± 0.2 hi
		150	27.3 ± 0.3 ghi	26.8 ± 0.3 ghi
		300	26.2 ± 0.3 hi	25.8 ± 0.5 i
	140	0	28.3 ± 0.2 d–h	26.8 ± 0.2 ghi
		150	29.3 ± 0.5 b–g	26.5 ± 0.2 ghi
		300	30.2 ± 0.3 bcde	27.6 ± 0.2 fgh
	160	0	29.5 ± 0.3 b–g	30.5 ± 0.3 cd
		150	30.6 ± 0.5 bc	29.5 ± 0.3 de
		300	30.1 ± 0.4 b–f	30.1 ± 0.3 cd
Large-Raw	-	-	31.1 ± 0.8 b	34.4 ± 0.3 b
Large-Extruded	120	0	25.9 ± 0.2 i	27.9 ± 0.1 efg
		150	27.9 ± 0.3 fghi	29.3 ± 0.1 def
		300	26.9 ± 0.3 hi	29.9 ± 0.1 d
	140	0	28.1 ± 0.1 e–i	28.9 ± 0.3 def
		150	28.4 ± 0.2 c–h	29.3 ± 0.2 def
		300	29.7 ± 0.7 b–f	31.6 ± 0.5 c
	160	0	28.4 ± 0.4 c–h	28.9 ± 0.6 def
		150	28.5 ± 0.4 c–h	30.4 ± 0.2 cd
		300	30.5 ± 0.5 bcd	31.7 ± 0.1 c

In each column, values designated with different letters are significantly (*p* ≤ 0.05) different. Values represent mean ± standard error (*n* = 3).

**Table 4:** Pasting properties of raw and extruded yellow pea flours as a function of flour particle size, die temperature (DT) and air injection pressure.

Flour Particle Size	DT	Air Pressure	Cold Viscosity	Peak Viscosity	Trough Viscosity	Final Viscosity
	(°C)	(kPa)	(cP)	(cP)	(cP)	(cP)
Small-Raw	-	-	36.0 ± 1.0	937.0 ± 14.0	888.5 ± 6.5	2093.0 ± 55.0
Small-Extruded	120	0	172.0 ± 2.4 ghi	-	91.5 ± 1.7 ef	259.0 ± 6.9 h
		150	186.8 ± 4.3 e-i	-	103.0 ± 3.3 def	287.0 ± 1.5 gh
		300	201.8 ± 7.6 defg	-	105.3 ± 4.2 cdef	300.3 ± 3.3 fgh
	140	0	195.3 ± 1.6 d-h	-	103.3 ± 2.6 def	301.0 ± 2.9 fgh
		150	223.3 ± 4.9 cd	-	92.8 ± 4.2 ef	304.3 ± 4.4 fgh
		300	236.8 ± 10.5 c	-	99.3 ± 4.8 ef	346.5 ± 12.5 ef
	160	0	208.5 ± 4.1 cdef	-	111.0 ± 4.9 cde	336.3 ± 13.2 efg
		150	176.3 ± 6.6 ghi	-	84.5 ± 2.4 fg	331.8 ± 7.7 efg
		300	166.0 ± 5.1 hi	-	68.8 ± 1.9 g	272.5 ± 12.1 h
Large-Raw	-	-	32.0 ± 1.0	857.0 ± 2.0	836.0 ± 0.0	1707.5 ± 8.5
Large-Extruded	120	0	182.0 ± 6.2 fghi	-	127.3 ± 9.3 bc	373.8 ± 18.7 e
		150	168.3 ± 7.4 hi	-	112.5 ± 3.4 cde	436.8 ± 7.8 cd
		300	161.0 ± 4.1 i	-	124.3 ± 2.8 bcd	456.3 ± 7.9 c
	140	0	221.3 ± 6.7 cd	-	168.5 ± 5.6 a	383.8 ± 4.6 de
		150	217.5 ± 7.7 cde	-	106.8 ± 2.7 cdef	433.0 ± 13.4 cd
		300	296.8 ± 7.5 b	-	142.5 ± 7.2 b	666.5 ± 25.0 a
	160	0	336.0 ± 1.8 a	-	184.3 ± 1.9 a	474.0 ± 4.9 c
		150	274.3 ± 7.1 b	-	145.0 ± 3.1 b	668.5 ± 9.6 a
		300	271.5 ± 7.5 b	-	138.0 ± 4.2 b	592.8 ± 13.1 b

Pasting properties are significantly (*p* ≤ 0.05) different if designated with different letters. Values represent mean ± standard error (*n* = 2 for extrusion and RVA viscosities).

## Data Availability

The data that support the findings of this study are available on request from the corresponding author.

## References

[1] Boye J., Zare F., Pletch A. (2010). Pulse proteins: Processing, characterization, functional properties and applications in food and feed. Food Res. Int..

[2] Mshayisa V.V., Van Wyk J., Zozo B. (2022). Nutritional, techno-functional and structural properties of black soldier fly (Hermetia illucens) larvae flours and protein concentrates. Foods.

[3] Kostić A.T., Barać M.B., Stanojević S.P., Milojković-Opsenica D.M., Tešić Ž.L., Šikoparija B., Radišić P., Prentović M., Pešić M.B. (2015). Physicochemical composition and techno-functional properties of bee pollen collected in Serbia. LWT-Food Sci. Technol..

[4] Vaclavik V.A., Christian E.W. (2014). Essentials of Food Science.

[5] Thakur S., Scanlon M.G., Tyler R.T., Milani A., Paliwal J. (2019). Pulse Flour Characteristics from a Wheat Flour Miller’ s Perspective: A Comprehensive Review. Compr. Rev. Food Sci. Food Saf..

[6] Scanlon M.G., Thakur S., Tyler R.T., Milani A., Der T., Paliwal J. (2018). The critical role of milling in pulse ingredient functionality. Cereal Foods World.

[7] Carvalho C.W.P., Takeiti C.Y., Onwulata C.I., Pordesimo L.O. (2010). Relative effect of particle size on the physical properties of corn meal extrudates: Effect of particle size on the extrusion of corn meal. J. Food Eng..

[8] Singh R., Koksel F. (2021). Effects of particle size distribution and processing conditions on the techno-functional properties of extruded soybean meal. LWT-Food Sci. Technol..

[9] Alonso R., Orue E., Zabalza M.J., Grant G., Marzo F. (2000). Effect of extrusion cooking on structure and functional properties of pea and kidney bean proteins. J. Sci. Food Agric..

[10] Luo S., Koksel F. (2020). Physical and technofunctional properties of yellow pea flour and bread crumb mixtures processed with low moisture extrusion cooking. J. Food Sci..

[11] Rocha-Guzman N.E., Gallegos-Infante J.A., Gonzalez-Laredo R.F., Bello-Perez A., Delgado-Licon E., Ochoa-Martinez A., Prado-Ortiz M.J. (2008). Physical properties of extruded products from three Mexican common beans (*Phaseolus vulgaris* L.) cultivars. Plant Foods Hum. Nutr..

[12] Bueno A.S., Pereira C.M., Menegassi B., Arêas J.A.G., Castro I.A. (2009). Effect of extrusion on the emulsifying properties of soybean proteins and pectin mixtures modelled by response surface methodology. J. Food Eng..

[13] Mozafarpour R., Koocheki A., Milani E., Varidi M. (2019). Extruded soy protein as a novel emulsifier: Structure, interfacial activity and emulsifying property. Food Hydrocoll..

[14] Lee E.Y., Ryu G.H., Lim S.T. (1999). Effects of processing parameters on physical properties of corn starch extrudates expanded using supercritical CO_2_ injection. Cereal Chem..

[15] Singkhornart S., Edou-Ondo S., Ryu G.H. (2014). Influence of germination and extrusion with CO_2_ injection on physicochemical properties of wheat extrudates. Food Chem..

[16] Li X., Masatcioglu M.T., Koksel F. (2019). Physical and functional properties of wheat flour extrudates produced by nitrogen injection assisted extrusion cooking. J Cereal Sci..

[17] Sinaki N.Y., Tulbek M., Koksel F. (2021). Oxidizing agent assisted extrusion cooking of yellow peas and the techno-functionality of the resulting extrudate flours. J. Food Process. Preserv..

[18] Luo S., Chan E., Masatcioglu M.T., Erkinbaev C., Paliwal J., Koksel F. (2020). Effects of extrusion conditions and nitrogen injection on physical, mechanical, and microstructural properties of red lentil puffed snacks. Food Bioprod Process..

[19] Ooms N., Delcour J.A. (2019). How to impact gluten protein network formation during wheat flour dough making. Curr Opin Food Sci..

[20] Joye I.J., Lagrain B., Delcour J.A. (2009). Use of chemical redox agents and exogenous enzymes to modify the protein network during breadmaking-A review. J Cereal Sci..

[21] Chaudhary A., Marinangeli C.P.F., Tremorin D., Mathys A. (2018). Nutritional combined greenhouse gas life cycle analysis for incorporating canadian yellow pea into cereal-based food products. Nutrients.

[22] Donihee L., McDougall T. (2022). Canada: Outlook for principal field crops. Agric. Agri-Food Can..

[23] Wang C., Alavi S., Li Y., Dogan H. (2022). Influence of chickpea flour and yellow pea concentrate additive amount and in-barrel moisture content on the physiochemical properties of extruded extrudates. J. Food Process. Preserv..

[24] AACC International (2000). Approved Methods of American Association of Cereal Chemists (AACC) Methods 44-01, 08-01, 30-25, and 46-30.

[25] Koksel F., Masatcioglu M.T. (2018). Physical properties of puffed yellow pea snacks produced by nitrogen gas assisted extrusion cooking. LWT-Food Sci. Technol..

[26] Masatcioglu T., Koksel F., Masatcioglu M.T., Koksel F., Masatcioglu T., Koksel F. (2019). Functional and thermal properties of yellow pea and red lentil extrudates produced by nitrogen gas injection assisted extrusion cooking. J. Sci. Food Agric..

[27] Ahmedna M., Prinyawiwatkul W., Rao R.M. (1999). Solubilized wheat protein isolate: Functional properties and potential food applications. J. Agric. Food Chem..

[28] Pulivarthi M.K., Nkurikiye E., Watt J., Li Y., Siliveru K. (2021). Comprehensive understanding of roller milling on the physicochemical properties of red lentil and yellow pea flours. Processes.

[29] Maskus H., Bourré L., Fraser S., Sarkar A., Malcolmson L. (2016). Effects of grinding method on the compositional, physical, and functional properties of whole and split yellow pea flours. Cereal Foods World.

[30] Onwulata C.I., Konstance R.P. (2006). Extruded corn meal and whey protein concentrate: Effect of particle size. J. Food Process. Preserv..

[31] Desrumaux A., Bouvier J.M., Burri J. (1998). Corn grits particle size and distribution effects on the characteristics of expanded extrudates. J. Food Sci..

[32] Al-Rabadi G.J., Torley P.J., Williams B.A., Bryden W.L., Gidley M.J. (2011). Particle size of milled barley and sorghum and physico-chemical properties of grain following extrusion. J. Food Eng..

[33] Mathew J.M., Hoseney R.C., Faubion J.M. (1999). Effects of corn sample, mill type, and particle size on corn curl and pet food extrudates. Cereal Chem..

[34] Meng X., Threinen D., Hansen M., Driedger D. (2010). Effects of extrusion conditions on system parameters and physical properties of a chickpea flour-based snack. Food Res. Int..

[35] Nwabueze T.U. (2006). Water/oil absorption and solubility indices of extruded African breadfruit (Treculia africana) blends. J. Food Technol..

[36] Bhattacharya M., Milford M.A. (1988). Extrusion processing to improve nutritional and functional-properties of corn gluten. LWT-Food Sci. Technol..

[37] Protonotariou S., Drakos A., Evageliou V., Ritzoulis C., Mandala I. (2014). Sieving fractionation and jet mill micronization affect the functional properties of wheat flour. J. Food Eng..

[38] Chauhan G.S., Bains G.S. (1985). Effect of granularity on the characteristics of extruded rice snack. Int. J. Food Sci. Technol..

[39] Zheng J., Mao S. (2019). A thermodynamic model for the solubility of N_2_, O_2_ and Ar in pure water and aqueous electrolyte solutions and its applications. Appl Geochem..

[40] Campbell G.M., Cauvain S.P. (2003). Bread aeration. Bread Making: Improving Quality.

[41] Decamps K., Joye I.J., De Vos D.E., Courtin C.M., Delcour J.A. (2016). Molecular oxygen and reactive oxygen species in bread-making processes: Scarce, but nevertheless important. Crit. Rev. Food Sci. Nutr..

[42] Wieser H., Cauvain S.P. (2003). The use of redox agents in breadmaking. Bread Making: Improving Quality.

[43] Huang Y., Hua Y., Qiu A. (2006). Soybean protein aggregation induced by lipoxygenase catalyzed linoleic acid oxidation. Food Res Int..

[44] Wu W., Zhang C., Kong X., Hua Y. (2009). Oxidative modification of soy protein by peroxyl radicals. Food Chem..

[45] Xiong Y.L., Guo A. (2021). Animal and plant protein oxidation: Chemical and functional property significance. Foods.

[46] Aluko R.E., Mofolasayo O.A., Watts B.M. (2009). Emulsifying and foaming properties of commercial yellow pea (*Pisum sativum* L.) seed flours. J. Agric. Food Chem..

[47] Beck S.M., Knoerzer K., Sellahewa J., Emin M.A., Arcot J. (2017). Effect of different heat-treatment times and applied shear on secondary structure, molecular weight distribution, solubility and rheological properties of pea protein isolate as investigated by capillary rheometry. J. Food Eng..

[48] Wang S., Nosworthy M.G., House J.D., Ai Y., Hood-Niefer S., Nickerson M.T. (2019). Effect of barrel temperature and feed moisture on the physical properties of chickpea–sorghum and chickpea–maize extrudates, and the functionality and nutritional value of their resultant flours—Part II. Cereal Chem..

[49] Wang S., Ai Y., Hood-Niefer S., Nickerson M.T. (2019). Effect of barrel temperature and feed moisture on the physical properties of chickpea, sorghum, and maize extrudates and the functionality of their resultant flours—Part 1. Cereal Chem..

[50] Martínez M.M., Calviño A., Rosell C.M., Gómez M. (2014). Effect of different extrusion treatments and particle size distribution on the physicochemical properties of rice flour. Food Bioprocess Technol..

[51] Tang C.H., Shen L. (2013). Role of conformational flexibility in the emulsifying properties of bovine serum albumin. J. Agric. Food Chem..

[52] Karaca A.C., Low N., Nickerson M. (2011). Emulsifying properties of chickpea, faba bean, lentil and pea proteins produced by isoelectric precipitation and salt extraction. Food Res. Int..

[53] Foschia M., Horstmann S.W., Arendt E.K., Zannini E. (2017). Legumes as functional ingredients in gluten-free bakery and pasta products. Annu. Rev. Food Sci. Technol..

[54] Afizah M.N., Rizvi S.S.H. (2014). Functional properties of whey protein concentrate texturized at acidic pH: Effect of extrusion temperature. LWT-Food Sci. Technol..

[55] Ma Z., Boye J.I., Simpson B.K., Prasher S.O., Monpetit D., Malcolmson L. (2011). Thermal processing effects on the functional properties and microstructure of lentil, chickpea, and pea flours. Food Res. Int..

[56] Sikorski Z.E., Sikorski Z.E., Strauss S. (2001). Functional properties of proteins in food systems. Chemical and Functional Properties of Food Proteins.

[57] Zeng J., Gao H., Li G., Liang X. (2011). Extruded Corn Flour Changed the Functionality Behaviour of Blends. Czech J. Food Sci..

[58] Wani S.A., Kumar P. (2019). Influence on the antioxidant, structural and pasting properties of snacks with fenugreek, oats and green pea. J. Saudi Soc. Agric. Sci..

[59] Tananuwong K., Malila Y. (2011). Changes in physicochemical properties of organic hulled rice during storage under different conditions. Food Chem..

